# *In Vitro* Persistence Level Reflects *In Vivo* Antibiotic Survival of Natural Pseudomonas aeruginosa Isolates in a Murine Lung Infection Model

**DOI:** 10.1128/spectrum.04970-22

**Published:** 2023-05-04

**Authors:** Laure Verstraete, Juliana Aizawa, Matthias Govaerts, Linda De Vooght, Rob Lavigne, Jan Michiels, Bram Van den Bergh, Paul Cos

**Affiliations:** a Centre of Microbial and Plant Genetics, KU Leuven, Leuven, Belgium; b Center for Microbiology, Flanders Institute for Biotechnology, Leuven, Belgium; c Laboratory for Microbiology, Parasitology and Hygiene, University of Antwerp, Antwerp, Belgium; d Laboratory of Gene Technology, KU Leuven, Leuven, Belgium; The University of North Carolina at Chapel Hill

**Keywords:** persistence, antibiotics, tolerance, *Pseudomonas aeruginosa*, *in vivo* model

## Abstract

Clinicians are increasingly confronted with the limitations of antibiotics to clear bacterial infections in patients. It has long been assumed that only antibiotic resistance plays a pivotal role in this phenomenon. Indeed, the worldwide emergence of antibiotic resistance is considered one of the major health threats of the 21st century. However, the presence of persister cells also has a significant influence on treatment outcomes. These antibiotic-tolerant cells are present in every bacterial population and are the result of the phenotypic switching of normal, antibiotic-sensitive cells. Persister cells complicate current antibiotic therapies and contribute to the development of resistance. In the past, extensive research has been performed to investigate persistence in laboratory settings; however, antibiotic tolerance under conditions that mimic the clinical setting remain poorly understood. In this study, we optimized a mouse model for lung infections with the opportunistic pathogen Pseudomonas aeruginosa. In this model, mice are intratracheally infected with P. aeruginosa embedded in seaweed alginate beads and subsequently treated with tobramycin via nasal droplets. A diverse panel of 18 P. aeruginosa strains originating from environmental, human, and animal clinical sources was selected to assess survival in the animal model. Survival levels were positively correlated with the survival levels determined via time-kill assays, a common method to study persistence in the laboratory. We showed that survival levels are comparable and thus that the classical persister assays are indicative of antibiotic tolerance in a clinical setting. The optimized animal model also enables us to test potential antipersister therapies and study persistence in relevant settings.

**IMPORTANCE** The importance of targeting persister cells in antibiotic therapies is becoming more evident, as these antibiotic-tolerant cells underlie relapsing infections and resistance development. Here, we studied persistence in a clinically relevant pathogen, Pseudomonas aeruginosa. It is one of the six ESKAPE pathogens (Enterococcus faecium, Staphylococcus aureus, Klebsiella pneumoniae, Acinetobacter baumannii, P. aeruginosa, and Enterobacter spp.), which are considered major health threats. P. aeruginosa is mostly known to cause chronic lung infections in cystic fibrosis patients. We mimicked these lung infections in a mouse model to study persistence under more clinical conditions. It was shown that the survival levels of natural P. aeruginosa isolates in this model are positively correlated with the survival levels measured in classical persistence assays *in vitro*. These results not only validate the use of our current techniques to study persistence but also open opportunities to study new persistence mechanisms or evaluate new antipersister strategies *in vivo*.

## INTRODUCTION

Pseudomonas aeruginosa is a Gram-negative, opportunistic pathogen commonly associated with chronic wound infections and airway infections in cystic fibrosis (CF) patients (1, 2). The intrinsic antibiotic resistance of P. aeruginosa and its ability to acquire additional resistance mechanisms make P. aeruginosa infections very challenging to treat (3). It is considered a priority pathogen by the World Health Organization for which new treatments are urgently needed and drug research should be prioritized (4). A recent analysis of the impact of antimicrobial resistance worldwide estimates that in 2019, more than 300,000 deaths were associated with antibiotic-resistant P. aeruginosa infections (5). Not only resistance but also the presence of a subset antibiotic-tolerant cells may explain the difficulty of eradicating P. aeruginosa infections (6, 7). Indeed, when this organism is challenged with a high dose of bactericidal antibiotics, a small fraction of persister cells are able to survive even in the absence of antibiotic resistance. Persister cells are phenotypic variants of normal antibiotic-sensitive cells that have transiently switched to a nongrowing, antibiotic-tolerant state. In the absence of antibiotics, these cells give rise to a new bacterial population that is as susceptible to antibiotics as the original population (8, 9). Due to their small fraction, persisters are often neglected or overlooked in clinical practice, but they are nonetheless of great clinical concern, as they are inherently present in all bacterial populations and underlie antibiotic therapy failure (10). Persisters are especially relevant in biofilms and intracellular infections and in immunocompromised patients, in whom the immune system is not able to eliminate all cells (10, 11). More recently, it was shown that persisters catalyze resistance development in several ways. Persisters form a pool of viable cells from which resistant mutants can emerge via *de novo* mutations (12, 13) or horizontal gene transfer (14). Moreover, higher persistence levels are associated with higher mutation rates conferring resistance in natural isolates and mutated lab strains of Escherichia coli (15). A positive correlation between resistance and persistence levels has also been observed in Pseudomonas isolates (16), suggesting a complementary link between the two survival strategies. Thus, targeting persister cells not only kills antibiotic-tolerant cells but also impedes resistance development. As its clinical importance is gradually being acknowledged, recent years have seen an increased interest in research on persistence. Most studies have focused on the mechanisms of persister formation (17) and awakening (18–20) in E. coli, but the complete picture has not been fully delineated. Also, knowledge on the physiology of persister cells of major pathogens such as P. aeruginosa remains fragmentary. Some of the previously established persister mechanisms are condition dependent (8) and have mainly been observed under lab conditions, which raises the question of whether the same mechanisms are relevant in real-life infections. Only a few studies could extrapolate *in vitro* findings on persistence to an *in vivo* setting (21–23), whereas others obtained mixed (24) or contradictory (25, 26) results. Current persistence models have focused primarily on lung infections caused by Mycobacterium species (26–29) and intracellular infections by Salmonella (14, 22, 25, 30–34). In the past, P. aeruginosa persisters were studied *in vivo* using an intraperitoneal and subcutaneous biofilm infection model in mice (21). However, P. aeruginosa is especially lethal when infecting the lungs (35), and an appropriate lung infection model to study antibiotic tolerance is, to our knowledge, not available. Moreover, it is unknown whether the number of persisters quantified via standardized persister assays in the lab resembles antibiotic tolerance *in vivo*. To fill this knowledge gap, we optimized a murine lung infection model with seaweed alginate beads to study antibiotic tolerance. Additionally, we assessed a diverse set of natural P. aeruginosa isolates in classical *in vitro* persister assays and compared the results to their survival in the murine lung model. We found that the survival level is highly variable among the strains both *in vitro* and *in vivo*. More importantly, the survival in the murine model positively correlates with the survival measured in laboratory settings. While some deviations between *in vitro* and *in vivo* findings were found, our results show that *in vitro* setups to study antibiotic tolerance are appropriate. Moreover, in future studies, the optimized infection model could be used to test new antipersister therapies to combat P. aeruginosa infections or to study tolerance mechanisms.

## RESULTS

### Optimization of the murine infection model.

**FIG 1:**
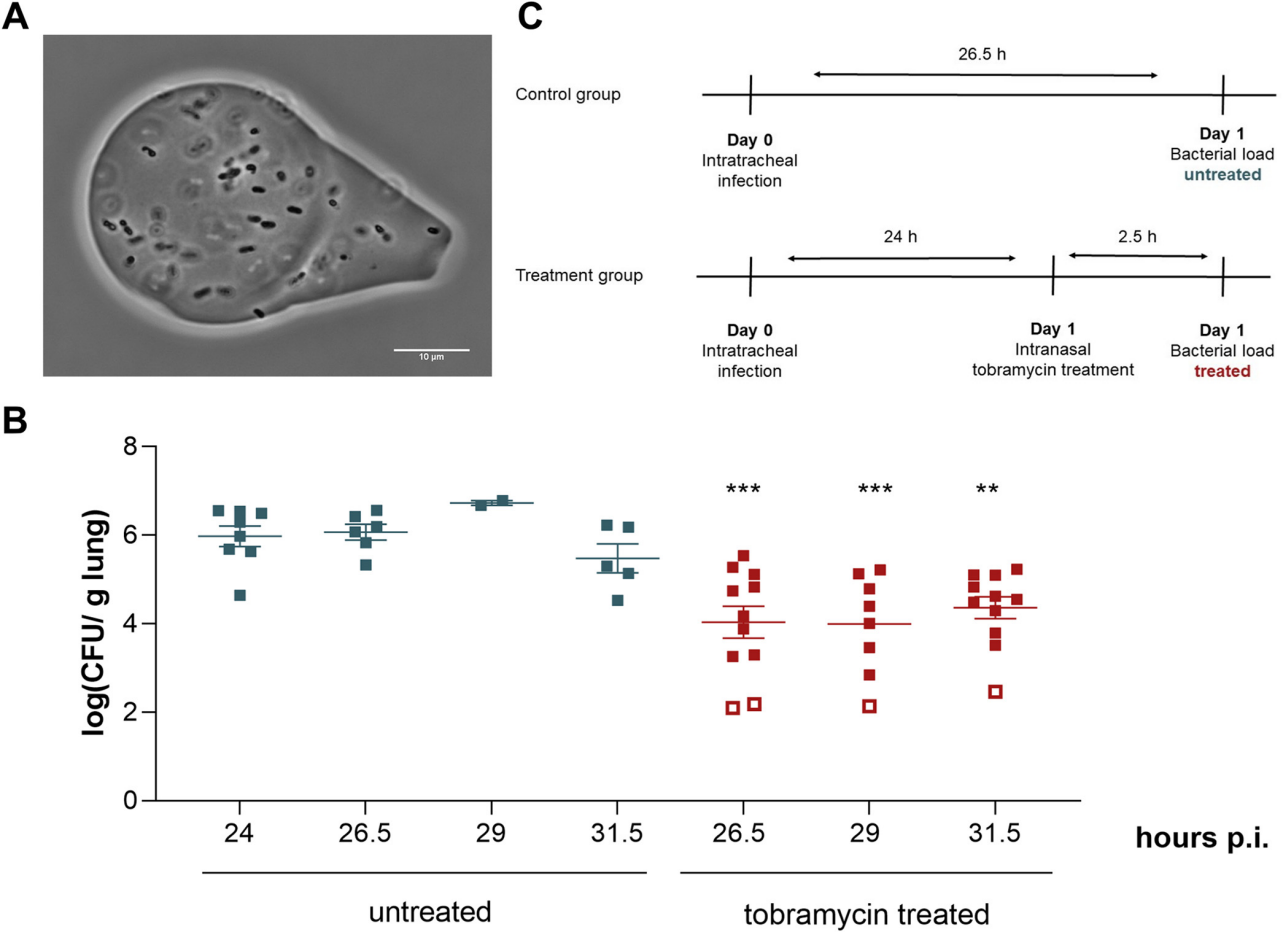
The lung infection model with P. aeruginosa embedded in seaweed alginate beads can be used to study antibiotic tolerance *in vivo*. (A) Microscopic image of seaweed alginate beads containing P. aeruginosa cells. (B) Results of a pilot experiment with PA14. The bacterial load in the lungs of untreated mice remained constant over time up to 31.5 h p.i. Upon tobramycin treatment at 24 h p.i., the number of cells in the murine lungs decreases significantly compared to that in the untreated mice at the same time point. Statistical differences between the bacterial load of untreated mice at 24 h p.i. and treated mice were determined with one-way ANOVA with a Dunnett’s *post hoc* test for multiple comparisons (**, *P* < 0.01; ***, *P* < 0.001). Each symbol represents one mouse, and the error bars show the standard errors of the means. Open squares indicate repeats below the detection limit, for which half of the detection limit divided by the lung weight is shown. (C) Optimized treatment scheme to determine the survival of antibiotic-tolerant cells in mice.

Mammalian species, and more specially rodents, are often the preferred animal model to study the *in vivo* pathogenesis of lung infections in a more controlled setting. Mice have been widely used in pulmonary research because of their small size and high reproducibility, the availability of inbred or transgenic strains, and the full characterization of mouse genetics and immunology (36, 37). To overcome the intrinsic tolerance of mice to human pathogens, the host immune system can be suppressed or physically separated from the bacteria. In this study, bacterial clearance by the murine immune system was prevented by infecting the mice with P. aeruginosa embedded in seaweed alginate beads (Fig. 1A). This infection model was first developed in rats with bacteria embedded in agar beads (38), adapted for mice (39), and further optimized with seaweed alginate beads (40) and in BALB/c mice (41, 42). The use of seaweed alginate beads mimics the later stages of chronic lung infections, where mucoid P. aeruginosa produces alginate, which is an important constituent of the extracellular matrix of biofilms (40, 43). Moreover, a study using agarose beads showed that the bacteria can still migrate from the beads and grow slowly, which is similar to the phenotype in biofilms (44). In our study, the seaweed alginate beads were directly installed in the lower respiratory tract of the mice via intratracheal instillation, which prevents animal injury and ensures the direct deposition of the inoculum, limiting variation between individual mice (45). After infection, mice were treated with high doses of tobramycin, an aminoglycoside antibiotic that is widely used for the treatment of P. aeruginosa infections. In patients, tobramycin is administered via inhalation therapy, in which the product is directly delivered in the airways (46, 47). Here, this antibiotic therapy was mimicked by the active inhalation of tobramycin via nasal droplets. Overall, these modifications result in a mouse model that mimics pulmonary infections in patients.

A first set of conditions was chosen based on the mouse model infected with the lab strain PA14. In a pilot experiment, BALB/c mice were intratracheally infected with several doses of PA14 embedded in seaweed alginate beads to study the infection course. Survival of the mice was measured 48 h postinfection (p.i.), and the bacterial load in the lungs was determined by plating and CFU enumeration. This dose-response experiment demonstrated that a challenge dose of 5 × 10^5^ CFU/mouse caused a stable infection and resulted in a bacterial titer of around 5 × 10^6^ CFU/g lung after 48 h (see Table S1 in the supplemental material). Higher doses resulted in a high mortality (50% to 100%), while at lower challenge doses all bacteria were cleared, as described earlier (44, 48, 49). It has been suggested that at higher inocula, the cytokine response is higher (44). We also hypothesize that at a lower inoculum, all bacterial cells are effectively targeted by the immune cells before a stable infection is established. For consistency, the same dose of 5 × 10^5^ CFU/mouse was used for all subsequent infections, including those with natural P. aeruginosa isolates. In the next step, the killing kinetics of bacteria in the lungs upon tobramycin treatment was characterized. A tobramycin dose of 120 mg/kg body weight, the highest tolerable dose, was applied after 24 h of infection with PA14-alginate beads. This tobramycin concentration, equivalent to 60 mg/mL, is at least 100,000-fold higher than the MIC of PA14 (~0.25 μg/mL), which should ensure that antibiotic-sensitive cells are effectively killed and antibiotic-tolerant cells survive. After antibiotic administration 24 h p.i., the bacterial load in the lung was measured every 2.5 h (Fig. 1B). After 2.5 h, the bacterial load of treated mice decreased 2 log compared to that in untreated mice. The killing rate slowed after 2.5 h, as the number of CFU in the lung did not further decrease over time. To check whether this CFU reduction is mainly caused by antibiotic exposure and not due to reduced survival in the lungs, the number of bacteria in the lungs of untreated mice was also measured. Indeed, the number of viable cells in untreated samples remained constant over the same period, indicating that the applied tobramycin dose effectively kills bacterial cells. The observed killing pattern upon treatment with high doses of antibiotics is consistent with biphasic killing kinetics, which is typically associated with antibiotic persistence (Fig. S1) (9). No dissemination of bacteria to the liver or spleen was observed (data not shown). In subsequent experiments using other P. aeruginosa strains, only one euthanasia time point was chosen for ethical reasons. Since no differences in CFU were observed between the early and late time points, it was decided to treat the mice for 2.5 h and thus kill both untreated and treated mice 26.5 h p.i. (Fig. 1C). An agar well diffusion assay demonstrated that the tobramycin concentration remained well above the MIC for PA14 throughout the treatment. The measured concentration of the homogenized lungs, sampled 2.5 h after treatment, was 8 ± 3 μg/g lung. These optimized conditions, i.e., challenge dose, tobramycin concentration, and treatment duration, were further used to determine the survival of natural isolates in mice.

### *In vitro* survival of natural P. aeruginosa isolates.

**FIG 2:**
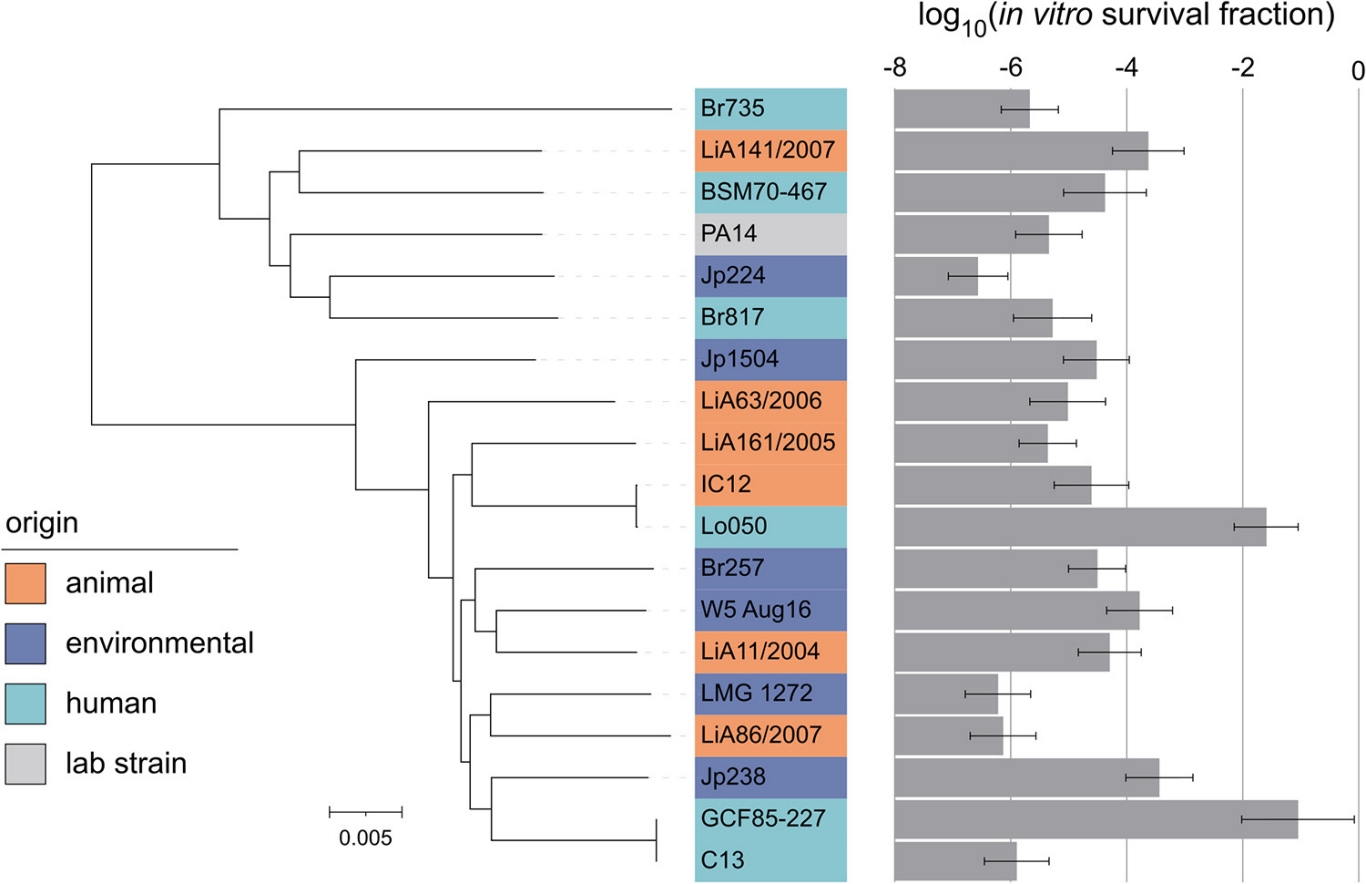
Phylogenetic tree based on the core genome of the P. aeruginosa strains used in this study. All isolates are colored according to their origin. The bar plot shows the *in vitro* survival based on the estimates of the biphasic kill curves (Table S2). The error bars show the 95% confidence intervals. PA7 was used as an outgroup to root the tree and is not shown. The scale bar indicates the number of substitutions per site.

**FIG 3:**
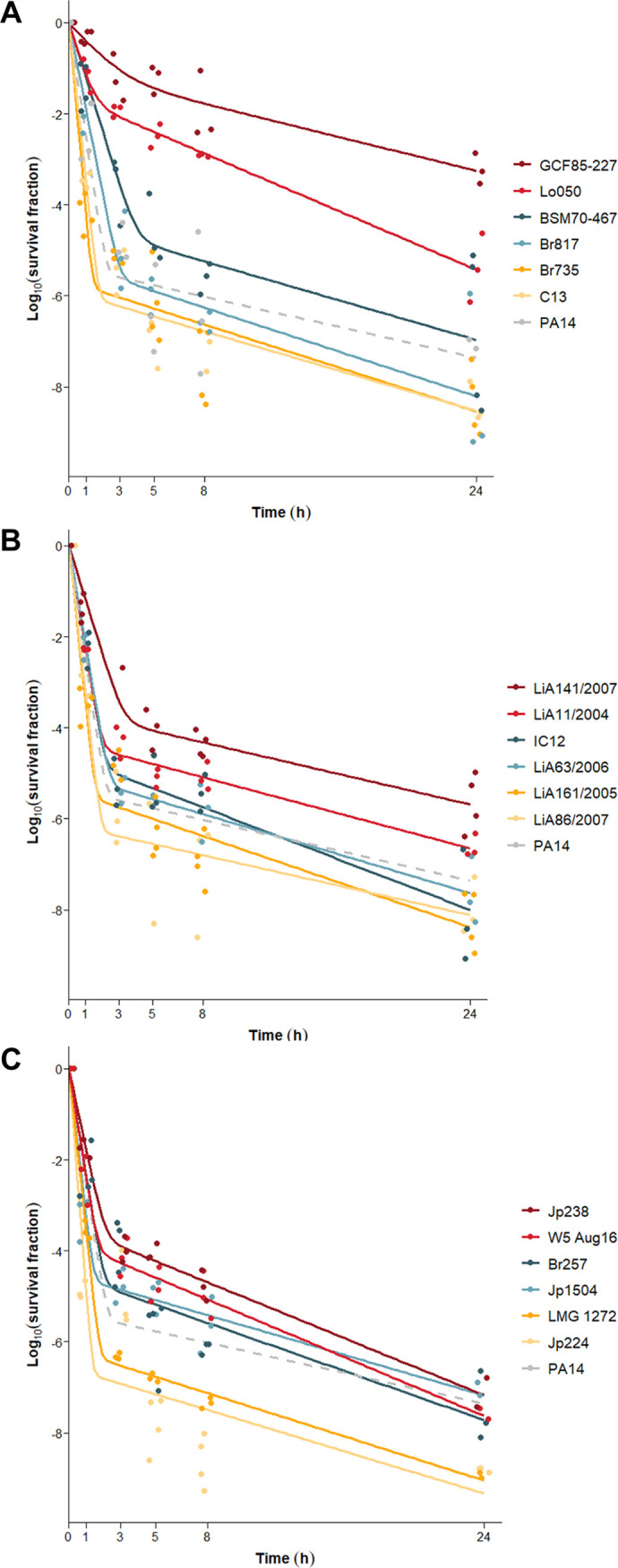
Time-kill curves of human (A), animal (B), and environmental (C) strains. Biphasic fittings of time-kill data in response to tobramycin treatment are shown in different colors according to their persistence phenotype; red, blue, and yellow indicate strains with high, intermediate, and low persistence levels, respectively, *in vitro*. Individual data points from at least three independent biological repeats are shown. The dotted line shows the killing kinetics of the lab strain PA14, which was included as a control in each experiment.

In a first series of tests, we selected a panel of natural P. aeruginosa isolates based on our *in vitro* findings that could later be used for testing in the animal model. In total, 18 isolates were chosen based on three main criteria: MIC, strain origin, and *in vitro* persistence level. To exclude antibiotic resistance as a confounding factor in bacterial survival, all strains were selected to be equally sensitive to tobramycin. The MICs of the selected isolates range between 0.125 and 0.5 μg/mL (Table 1). To capture the diversity within the species P. aeruginosa, these isolates were derived from diverse sources (Table 1). The final strain selection, therefore, includes human clinical strains (isolated from CF patients and wound infections), animal strains (isolated from dogs, kangaroos, parrots, and cats), and environmental strains (isolated from water sources and plants). The phylogenetic tree of the isolates, constructed based on the core genome, showed that strains from the same origin are not phylogenetically related, as they do not appear in the same clades, demonstrating the diversity of the strain collection (Fig. 2). Finally, the persistence levels vary across the selected isolates, which increases the statistical power of the comparison between the *in vitro* and *in vivo* survival. Time-kill experiments were performed to determine the persistence level of each P. aeruginosa isolate (Fig. 3). In these experiments, cultures were exposed to high doses of tobramycin (100× MIC) for several hours. The total numbers of cells observed at the start of the treatments were similar between strains (Fig. S2). To obtain a quantitative measure for persistence, the persister fractions were obtained from the estimated parameters of the biphasic models fitted to the time-kill data (Table S2). The persister fractions varied largely among the selected strains (*P* < 0.0001), ranging from 3 × 10^−7^ to 0.09, and were not phylogenetically correlated (Fig. 2). Human strains showed significantly higher survival levels than animal or environmental strains (*P* < 0.001). Strains could be categorized into three groups based on their *in vitro* survival fraction (Table 1). Strains were ordered from the lowest to the highest survival fraction. The first six and last six strains are considered strains with a low- and high-survival phenotype, respectively. The other six strains, those with an intermediate survival fraction, belong to the intermediate group. The average persister fraction between these three survival groups is significantly different (*P* < 0.0001) (Fig. S3A). In conclusion, a panel of diverse P. aeruginosa strains with low resistance levels was assembled. Moreover, a wide diversity in persistence levels between the strains was considered, which makes it suitable for comparison with survival *in vivo*.

**TABLE 1:** Natural P. aeruginosa isolates used in this study

Strain		Tobramycin MIC (μg/mL)	Survival phenotype		Origin	
Type	Name		*In vitro*	*In vivo*	Source	Country
Human	C13	0.25	Low	Low	CF patient	Germany
	BSM70-467	0.25	Intermediate	High	CF sputum	Belgium
	GCF85-227	0.125	High	High	CF sputum	Belgium
	Br817	0.25	Intermediate	Intermediate	Wound	Belgium
	Lo050	0.5	High	High	Burn	United Kingdom
	Br735	0.25	Low	Intermediate	Burn wound	Belgium

Animal	LiA86/2007	0.25	Low	Intermediate	Dog uterus	Portugal
	IC12	0.25	Intermediate	High	Dog	India
	LiA63/2006	0.25	Intermediate	Low	Kangaroo blood	Portugal
	LiA141/2007	0.25	High	High	Dog eye	Portugal
	LiA161/2005	0.25	Low	Low	Parrot eye	Portugal
	LiA11/2004	0.25	High	Intermediate	Cat nose	Portugal

Environmental	Jp224	0.5	Low	Low	Sea water (open ocean)	Japan
	Jp1504	0.5	Intermediate	High	River water	Japan
	W5 Aug16	0.25	High	Intermediate	River water	Belgium
	LMG 1272	0.25	Low	Low	Mushroom	United Kingdom
	Br257	0.25	Intermediate	Low	Plant rhizosphere	Belgium
	Jp238	0.25	High	Intermediate	Sea water (coastal)	Japan

### *In vivo* survival of natural P. aeruginosa isolates.

The representative strain collection was used to study the survival upon antibiotic exposure in the optimized murine model that mimics P. aeruginosa lung infections. In a first set of experiments, the optimized challenge dose of 5 × 10^5^ CFU/mouse was tested for all 18 isolates. All mice survived 26.5 h after infection with this dose (Fig. S4A), and dissemination to liver and spleen was limited (Fig. S4B and C). This challenge dose could thus be used for subsequent treatment experiments. In these experiments, mice were treated at 24 h p.i. with tobramycin (120 mg/kg mouse) via nasal droplets and sacrificed 2.5 h later. Euthanasia of untreated mice (control group) was performed at 26.5 h p.i. to make the infection process comparable to that of treated mice (Fig. 1C). Although the inoculum dose was the same for all strains, the number of cells in untreated mice at 26.5 h p.i. varied between strains (Fig. S5), showing differences in *in vivo* fitness between strains. Mice treated with tobramycin showed a significant reduction of bacteria in the lungs compared to the control group, with an average reduction across all infected isolates of 1 log (*P* = 0.02; data not shown). To enable comparison between *in vitro* survival and *in vivo* survival, a similar quantitative measure was calculated. The *in vitro* persister fraction is defined as the ratio of the number of surviving bacteria after treatment to the number of viable bacteria before treatment. In this study, the *in vivo* survival fraction in one mouse is defined as the number of cells after treatment in the left lung of that mouse divided by the average number of cells in the left lungs of all untreated mice at 26.5 h p.i.:

(1) $$\mathrm{in}\;\mathrm{vivo}\text{ survival fraction}=\frac{\frac{\text{CFU}}{\text{g lung}}\text{treated}}{{\text{10}}^{{\text{average(log}}_{\text{10}}\left(\frac{\text{CFU}}{\text{g lung}}\text{untreated}\right)\text{)}}}$$

**FIG 4:**
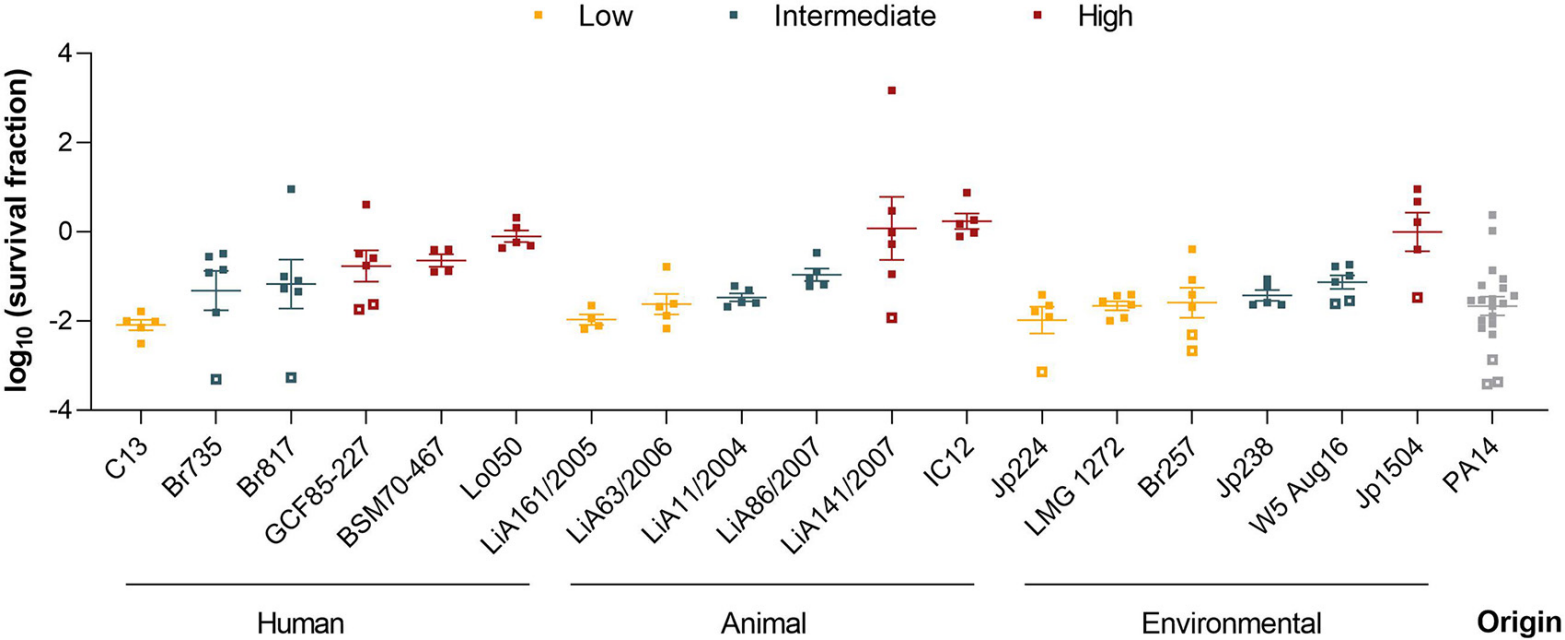
*In vivo* survival of P. aeruginosa isolates. Strains are sorted according to their origin and colored according to their *in vivo* survival phenotype, with yellow, blue, and red representing low, intermediate, and high survival levels, respectively. Each square represents the survival fraction of individual mice observed in the murine lung infection model, calculated as described in reference 1. Open squares indicate treated samples below the detection limit. Error bars represent the standard errors of the means.

Analyses similar to those for the *in vitro* survival fractions were performed on these calculated *in vivo* survival fractions. The observed *in vivo* survival fractions are variable among the different isolates (*P* < 0.0001) (Fig. 4). However, in contrast to *in vitro* survival, there is no significant effect of the strain’s origin on the *in vivo* survival (*P* = 0.23). The isolates were again categorized into three groups based on their *in vivo* survival: low, intermediate, and high (Table 1). The average survival fraction between these three groups is significantly different (*P* < 0.0001) (Fig. S3B). Remarkably, the survival fraction upon antibiotic treatment is negatively correlated with the bacterial counts of untreated mice at 26.5 h p.i. (Fig. S6). This implies that the *in vivo* fitness of strains belonging to the low-survival group is higher than that of strains belonging to the high-survival group.

To assess possible changes in host immunological responses upon antibiotic treatment and their impact on the observed bacterial survival levels, the expression of a selection of cytokines and chemokines in the lungs was examined via RT-qPCR (Fig. S7). The expression levels of eight cytokines and chemokines were measured: interleukin-1β (IL-1β), tumor necrosis factor-α (TNF-α), IL-6, IL-10, keratinocyte chemoattractant (KC), macrophage inflammatory protein 2 (MIP-2), interferon gamma-inducible protein 10 (IP-10), and monocyte chemoattractant protein 1 (MCP-1). More specifically, the gene expression before and after treatment in response to a selection of the P. aeruginosa isolates with a diverse *in vivo* survival level was examined: LMG 1272, LiA63/2006, and Br257 (low survival level), Jp238 and Br817 (intermediate survival level), and Lo050 and LiA141/2007 (high survival level), with the lab strain PA14 included as control. In infected mice, the expression of IL-1β and KC was most upregulated. The effect of the lung infection seemed to be more moderate for the other cytokines (Fig. S7). Tobramycin treatment did not significantly change the expression of any of the cytokines tested, except for IP-10 (*P* = 0.02). The level of this proinflammatory chemokine was significantly increased in treated mice infected with strains Br817 (*P* = 0.01) and LiA141/2007 (*P* < 0.0001). We also studied whether there was a difference in immune response between strains belonging to a different survival group (Fig. S8). Mice challenged with high-survival strains demonstrated higher expression levels of KC and MCP-1 than those with strains belonging to the low- and intermediate-survival groups (*P* < 0.0001 for KC and MCP-1). In contrast, expression of the chemokine MIP-2 was highest in the low-survival group (*P* = 0.04). For other cytokines, expression levels were similar among all survival groups. Overall, these mouse experiments point to a diversity in survival among natural isolates upon tobramycin treatment, similar to what is observed *in vitro*. In the next step, survival *in vivo* was directly compared to survival *in vitro*.

### The antibiotic survival in lab and clinical settings is positively correlated.

**FIG 5:**
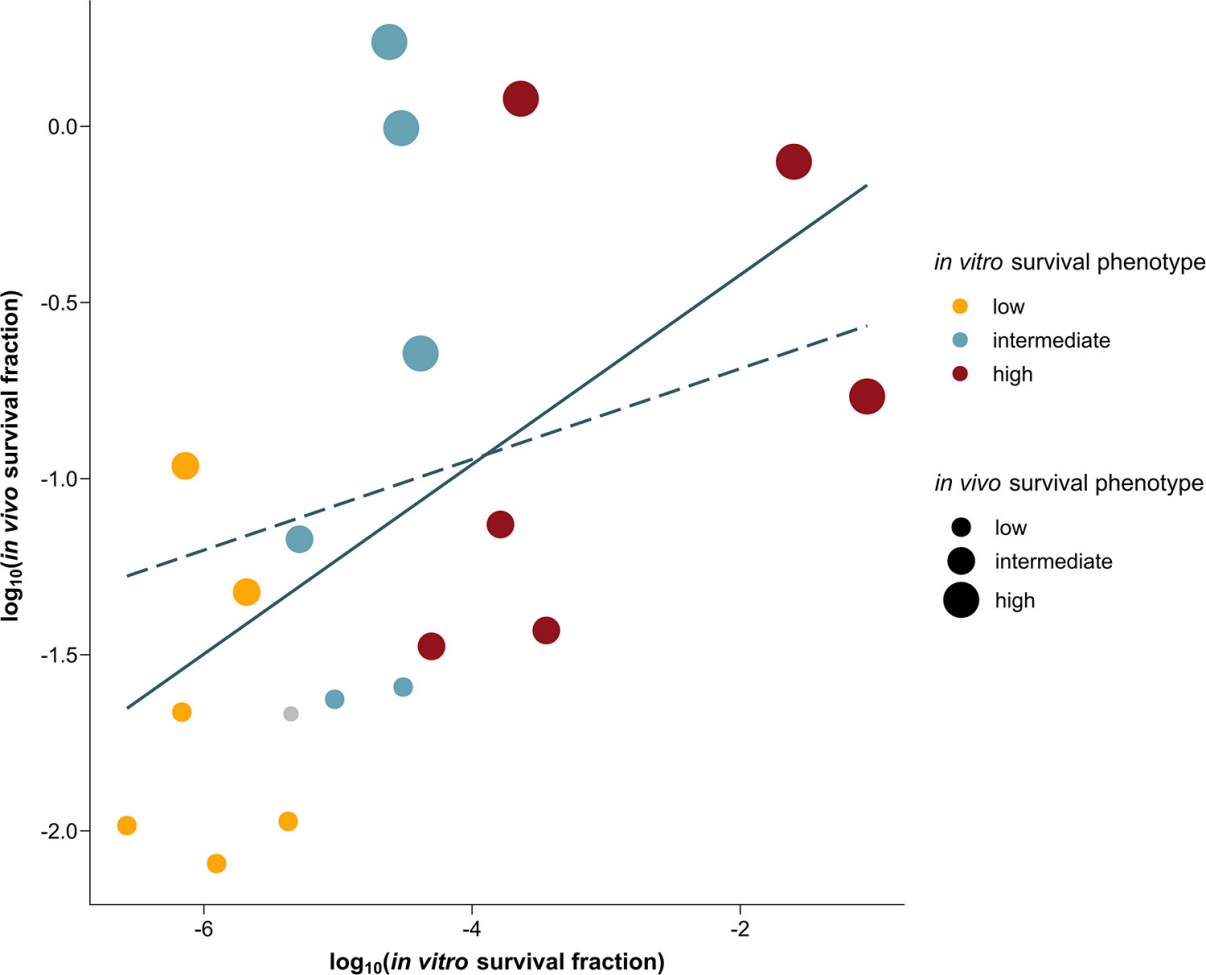
The *in vitro* survival is predictive of the *in vivo* survival. The mean survival fraction in both conditions is shown for all tested P. aeruginosa isolates. The 18 natural isolates are colored according to their phenotype *in vitro*; the size of the dots indicates their phenotype *in vivo*. The lab strain PA14 is represented by the gray dot. The solid line is the OLS regression line, and the dashed line is the PGLS regression line.

The 18 P. aeruginosa isolates can be ranked and categorized into three phenotype groups based on their *in vitro* and *in vivo* survival levels: low, intermediate and high (Table 1). Eight isolates are in the same phenotype groups *in vivo* and *in vitro*. The other 10 strains switched from a low-persister or high-persister type *in vitro* to an intermediate type *in vivo*, and vice versa. This indicates that there are only small differences in categorization based on survival, since strains did not switch phenotype groups or changed to only one group higher or lower. To further investigate the relationship between *in vitro* and *in vivo* survival, a phylogenetic generalized least-squares (PGLS) model was used to correct for nonindependence between the isolates. First, the phylogenetic signal of the data set was quantitatively measured via Pagel’s λ and Blomberg’s κ. A λ or κ value of 1 illustrates a strong phylogenetic signal in the survival levels, and a λ or κ value of 0 illustrates no phylogenetic signal (50). Both metrics were close to zero, which indicates a low, nonsignificant phylogenetic signal under a Brownian model of evolution. This implies that closely related isolates do not have more similar survival levels than distant isolates. However, it is difficult to estimate the phylogenetic signal for small data sets, and a low λ or κ does not necessarily mean that there is no phylogenetic signal (51–54). We performed and compared two extreme models with fixed κ values and λ values of 1 and 0 (Fig. 5; Table S3). The latter PGLS model is equivalent to an ordinary least-squares (OLS) model. Both models revealed a strong dependency of the *in vivo* survival on the *in vitro* survival. The *R*^2^ of the OLS model and PGLS model is 0.24 (*P* = 0.019) and 0.25 (*P* = 0.013), respectively, which means that around 25% of the variation in *in vivo* survival can be explained by *in vitro* survival levels. Overall, our data suggest that the survival observed in laboratory settings is comparable to survival under conditions that mimic the clinical situation.

## DISCUSSION

Since its discovery in the 1940s by Hobby et al. (55) and Bigger (56), persistence has gained great interest due to its role in relapsing infections (10) and resistance development (12–15). This eventually led to the publication of a consensus statement in which a clear definition of persistence and general guidelines to measure persistence are provided (9). One of the hallmarks to study persistence *in vitro* is the time-kill assay, in which bacterial survival upon exposure to high antibiotic concentrations is followed over time. However, to date, it is not clear whether the results of these time-kill assays reflect the antibiotic tolerance observed in patients. In this study, we have developed a murine lung infection model which enables us to study the *in vivo* tolerance of natural P. aeruginosa strains. Previous *in vivo* studies in persistence research mainly focused on lab strains and persistence mutants with higher or lower persistence levels, but natural isolates have, to our knowledge, never been studied in *in vivo* persistence research. We found that *in vitro* survival can partly predict the survival in an *in vivo* setting and thus validates the use of time-kill assays to quantify persistence.

In the *in vitro* experiments, all P. aeruginosa isolates across all origins showed a rapid decrease of the majority of the cells with only a small fraction of cells surviving the antibiotic treatment. We observed a large variation in persistence levels in natural isolates, similar to what was reported previously (15, 57–60). Remarkably, the number of surviving cells was higher in clinical than environmental strains. Variation in persister formation among isolates may be explained by their evolutionary history (8). The history of exposure to antibiotics or other environmental stresses of the examined isolates is unknown. Nevertheless, antibiotics, known inducers of persistence, in natural environments are in most cases present in lower concentrations than in patients, even lower than the MIC (61). It could be argued that strains isolated from clinical sources, such as CF lungs, experience higher selection pressure than strains isolated from nonclinical sources due to a prolonged exposure to high antibiotic doses. A similar effect clearly underlies the high persistence of late isolates from long-term-treated CF patients (6, 7). Moreover, isolates from chronic P. aeruginosa infections show increased tolerance compared to isolates from acute infections (62).

We established a lung infection model in mice to study the survival of multiple P. aeruginosa isolates. Although there are some important anatomical and physiological differences between murine lungs and human lungs, this chronic infection model with bacteria embedded in agar, agarose, or seaweed alginate beads is widely used to study the *in vivo* pathogenesis of lung infections and test the efficacy of new antibacterial therapies (63). In acute-infection models, free-living P. aeruginosa cells are directly administered to the murine lungs, resulting in rapid clearance of the cells or acute sepsis and eventually death (43). By embedding the bacteria in beads, bacterial clearance is avoided and the infection can be maintained for several weeks because there is a continued mild induction of the host immune system. Furthermore, the chronic model shows similarities with human pathology of chronic CF infections, including the histopathology and the increase in lung neutrophils and cytokines such as IL-1β, TNF-α, MIP-2, IL-6, and KC (40, 42, 44). The seaweed alginate bead model demonstrates a more pronounced antibody response than the agar bead model, which is also typical of chronic P. aeruginosa infections. Moreover, such alginate beads are often preferred because they mimic mucoid P. aeruginosa biofilms, where alginate is a constituent of the extracellular matrix (40).

In patients, antibiotic-tolerant biofilm structures make chronic infections very challenging to completely eradicate. The ability of biofilms to tolerate antibiotics has been attributed to several mechanisms, including subpopulations with differences in metabolic activity, the presence of persisters, differential gene expression, and limited diffusion of antibiotics through the biofilm matrix (64, 65). The latter mechanism appears to be particularly relevant for aminoglycosides, since these positively charged antibiotics bind to the negatively charged extracellular matrix of biofilms. We provide several arguments to demonstrate that tobramycin effectively penetrates the alginate beads in our murine model. Cao et al. (66) demonstrated that tobramycin penetrates large seaweed alginate beads *in vitro* and even accumulates in the alginate matrix. Also, several hours after removal of tobramycin from the beads, the bacteria inside the beads were still effectively killed, which shows that the concentration of free unbound tobramycin remains high (66). More detailed pharmacokinetic analyses reveal that the tobramycin concentration in the alginate matrix follows a power law as a function of the external concentration, probably by nonspecific binding to the matrix (67). These calculations should be interpreted with caution, as this power law dependence was calculated with *in vitro* alginate beads. Recently, Christophersen et al. (68) tested these findings in a chronic lung infection model in mice in which they followed the tobramycin concentration and bacterial killing in the alginate beads over time. This study confirmed the slow release of tobramycin from the alginate beads. Unbound tobramycin was detected several hours after tobramycin administration at low concentrations. It needs to be mentioned that direct comparison with our experiments is not possible, as tobramycin was administered subcutaneously at a lower dose in those experiments. In all the above-mentioned studies, the alginate density was also kept constant at 3% and larger alginate beads with diameters ranging from 4 to 5 mm were tested. Our alginate beads contain 1% alginate and have an average diameter of 25 μm (data not shown), which is more similar to the colony size observed in *in vivo* biofilms (69). Based on these arguments, we expect less retardation of diffusion through the beads in our optimized model. However, more elaborate pharmacokinetic and pharmacodynamic analyses are needed to determine the effective antibiotic concentration inside the alginate beads. Also, tobramycin is not completely degraded during the course of the experiment, as pharmacokinetic profiles of tobramycin in an acute infection model showed that the concentration in the murine lungs remains high for at least 24 h after internasal administration (70). Taking all these observations together, we believe that the administration of tobramycin for 2.5 h at an extreme high dose was sufficient to target all sensitive P. aeruginosa cells in the lungs.

In our lung infection model, tobramycin treatment significantly reduced the number of bacteria in the lungs. For all treatment durations, we observed a bacterial killing of PA14 of 2 log compared to the untreated group. Complete eradication upon tobramycin treatment in the seaweed alginate model was not observed, which is consistent with previous research (68, 70, 71). The timing of treatment probably plays an important role, as total clearance of bacteria in the lungs is observed when antibiotics are administered immediately after infection (70, 71). In the natural isolates, a less pronounced, but significant, reduction in CFU counts after treatment was observed. Although we also saw a significant difference in survival level between the isolates, this variation was less pronounced than in *in vitro* assays. Other factors may play a role in the observed tolerance of bacteria in chronic infections. In biofilms, cells undergo physiological and genetic alterations to survive in their new environment, which affects antibiotic tolerance as well (64, 65). In contrast to *in vitro* results, an effect of the strain’s origin on its *in vivo* survival was not observed. Bragonzi et al. demonstrated that CF strains and environmental P. aeruginosa strains have the same capacity to establish a chronic infection in murine lungs (72), but the effect of antibiotic treatment on strains with a different origin has not been demonstrated before. We also observed significant differences in *in vivo* fitness between strains, as was also demonstrated earlier in murine models of P. aeruginosa (42, 71). This might indicate differences in metabolic activity *in vivo* and/or in adaptive capacities for establishing a stable lung infection. These differences in fitness were not observed *in vitro*, as all strains were in stationary phase at the start of the treatment and CFU counts were comparable. Correlation analyses showed that the differences in *in vivo* fitness strongly impact the observed survival fractions *in vivo*. Indeed, higher CFU counts were obtained in mice infected with strains from the low-survival group. Nevertheless, the strongest reduction in CFU count upon antibiotic treatment was observed in these low-persister strains.

The cytokine profile revealed that upon tobramycin treatment, no change in inflammatory response was detected, which was also described earlier for tobramycin treatments starting later after infection (70). Remarkably, increased KC and MCP-1 levels before and after treatment were observed in high-survival strains. While investigating this was beyond the intended scope of this study, it could hint at a direct relationship between virulence, which was reflected here by changes in immunological responses, and antibiotic tolerance. A possible link between virulence and persistence has been proposed previously (73). Indeed, the stringent response, quorum sensing, and toxin-antitoxin modules play an important role in persistence and also regulate the expression of virulence factors. The relationship between virulence and antibiotic tolerance could be an interesting topic for further investigation. For example, investigating the *in vivo* expression levels of virulence factors, such as elastase, protease A, and pyocyanin, in strains with a variable tolerance level would be more informative than cytokine and chemokine expression levels. It is important to note that the increase in cytokine expression levels in our experiments seems only moderate. In future experiments, the inflammation in uninfected mice should be considered as well to allow comparison with normal expression levels of cytokines.

In this study, we used a murine model that mimics the later stages of chronic lung infections of P. aeruginosa. In humans, chronic lung infections are maintained by the production of a polysaccharide matrix, resulting in the retention of bacteria in the airways. Similarly, by embedding the bacteria in seaweed alginate beads, the clearance of bacteria is prevented. However, under natural conditions, respiratory infections in CF patients are characterized by colonization with multiple species, such as Staphylococcus aureus and Haemophilus influenzae, and P. aeruginosa appears only at a later stage (63). This colonization process was not mimicked here and could be tested in future research by infecting mice with different bacterial species over time. Indeed, previous research has shown that *in vivo* antibiotic tolerance is impacted by quorum-sensing molecules from other species (22). Instead of encapsulation of the pathogens, antibiotic tolerance could also be studied in CF mouse models in which the *Cftr* gene is knocked out. However, most CF models do not fully mimic typical lung diseases observed in CF patients (43). Another alternative model for studying antibiotic persistence in a more natural setting is a ventilator-associated pneumonia (VAP) model (74), in which mice are infected via endotracheal tubes.

Despite the distinct characteristics between *in vitro* and *in vivo* setups, we observed a correlation between the survival levels in the two systems. We are aware that the optimized model still has limitations and that the observed survival level cannot be explained solely by antibiotic tolerance. Nevertheless, strains with an extreme persistence phenotype have an extreme tolerance phenotype *in vivo* as well. For the first time, antibiotic tolerance of natural isolates between *in vitro* and *in vivo* setups was directly compared. Only one previous study on P. aeruginosa confirmed the *in vivo* tolerance phenotype of a Δ*relA ΔspoT* mutant upon ofloxacin treatment (21). Inactivation of both genes disrupts the stringent response and results in lower persistence levels than in the wild type *in vitro*. Intraperitoneal infection with the mutant increased mouse survival by 40% compared to the wild type after antibiotic treatment, which confirms the role of the stringent response in antibiotic tolerance (21). Other murine models in persistence research were focused on testing the efficacy of a new antipersister therapy (for examples, see references 75–78), unravelling the role of host factors (for examples, see references 28, 30, and 79), or tracking the metabolic activity with reporters for bacterial growth (for examples, see references 25, 28, and 31). The model described in this study allows us to both evaluate new drugs and study tolerance in a clinically relevant animal model.

## MATERIALS AND METHODS

### Bacterial strains and culture conditions.

Experiments were performed with natural P. aeruginosa strains isolated from clinical, environmental, and animal sources (Table 1). The lab strain UCBPP-PA14 (PA14) was used to optimize the *in vivo* model and included in each subsequent experiment. For the *in vitro* experiments, bacterial cultures were grown at 37°C in Mueller-Hinton broth (MHB; BD Difco) with orbital shaking or on lysogeny broth (LB; VWR International) agar. For the preparation of the seaweed alginate beads, P. aeruginosa strains were grown at 37°C in tryptic soy broth (TSB; BD Difco) with orbital shaking (150 rpm).

### Production of P. aeruginosa embedded in seaweed alginate beads.

The encapsulation of P. aeruginosa in seaweed alginate beads was performed as previously described with minor modifications (42, 80). One colony of the P. aeruginosa strain to be tested was incubated for 18 h in 50 mL of TSB at 37°C. The overnight bacterial culture was then centrifuged at 4,750 rpm for 10 min at 4°C, and the pellet was resuspended in 5 mL TSB. Six hundred microliters of this bacterial culture was suspended in 12 mL of sterile 1% alginate (FMC Biopolymer). The P. aeruginosa*-*alginate suspension was then transferred to a 20-mL syringe (BD Plastipak) fixed to a syringe pump (Multi-Phaser; ProSense) which was connected to an encapsulation unit (Var J30; Nisco). The suspension passed through the nozzle at a flow rate of 12 mL/h into a gelling bath. This gelling bath contained 0.1 M Tris-HCl buffer and 0.1 M CaCl_2_ (pH 7; Sigma) and was continuously mixed by magnetic stirring to avoid merging of the alginate beads. After 1 h of stirring in the gelling bath, the alginate beads were washed twice with 0.9% NaCl containing 0.1 M CaCl_2_ and suspended in this NaCl buffer. To determine the number of bacteria inside the beads, the alginate beads were dissolved using 0.5 M citrate buffer, serially diluted, plated on LB agar plates, and incubated for 24 h. The concentration of bacteria in the beads was approximately 10^7^ to 10^8^ CFU/mL. The alginate beads were visualized by phase-contrast microscopy using a Nikon Eclipse Ti-E inverted microscope equipped with a Qi2 CMOS camera.

### Determination of the MIC.

The MIC of an antibiotic is the lowest concentration at which the growth of the bacterial strain is inhibited and indicates the susceptibility of a strain to this antibiotic. To determine the MIC of tobramycin (TCI) of the isolates, the broth microdilution in 96-well microtiter plates or the agar dilution method was performed (81). For the broth microdilution method, an overnight culture was diluted in MHB to approximately 5 × 10^5^ cells/mL and incubated in a 2-fold dilution series of tobramycin at 37°C for 24 h. The MIC was determined by measuring the optical density at 595 nm with a Synergy Mx microplate reader (BioTek). For the agar dilution method, Mueller-Hinton agar plates were prepared with a concentration ranging from 0.0313 μg/mL to 64 μg/mL, with antibiotic-free plates as a positive control. An overnight culture was adjusted to a cell density of approximately 10^6^ CFU/mL, and subsequently, 10 μL of the suspension was spotted on the agar plates, resulting in a final inoculum of 10^4^ CFU per spot. Plates were incubated at 37°C for 20 to 24 h, after which the MIC was determined by visual inspection of colony formation. The MIC for each strain was determined based on at least two independent biological repeats.

### *In vitro* killing assays.

Strains were inoculated in 500 μL MHB in deep-well plates (U bottom) and incubated at 37°C for 24 h on a Titramax 1000 shaker (Heideloph) at 1,200 rpm. These cultures were then diluted 1:100 in fresh MHB medium and incubated under the same growth conditions. After 16 h, the cultures were diluted 1:2 in fresh medium, after which an aliquot of 200 μL was treated with tobramycin (100× MIC, concentration adjusted per strain). After 1, 3, 5, 8, and 24 h of incubation, the bacterial cells were washed twice with 10 mM MgSO_4_ to remove remaining antibiotics. The numbers of CFU before and after antibiotic treatment were determined by plating on LB agar. The plates with untreated cultures were counted after 24 h of incubation at 37°C, and plates with treated cultures were counted after 48 h.

### *In vivo* killing assays.

All animal experiments were authorized and approved by the Ethical Committee of the University of Antwerp (approval number 2018-91). Two hundred fifty-two female BALB/c mice, 12 weeks old, (Janvier Labs) were managed in accordance with the guidelines provided by the European Directive for Laboratory Animal Care (directive 2010/63/EU of the European Parliament). Mice were briefly sedated with isoflurane (Halocarbon), held in an upright position, and then intratracheally infected by pipetting the seaweed alginate beads with P. aeruginosa above the vocal cords. The optimized challenge dose was 5 × 10^5^ CFU/mouse. For this procedure, 40 μL of the alginate solution, diluted to the appropriate dose, was administered to each mouse. After 24 h of infection, a group of mice received one dose of approximately 120 mg/kg body weight tobramycin via intranasal application. Mice were held in a supine position, and 20 μL of tobramycin solution was administered in each nostril. Upon regaining consciousness, mice had unlimited access to feed and water. Body weight was monitored daily, since mice that lose ≥20% of their body weight are considered moribund and must be euthanized. Both untreated and treated mice were sacrificed at 24 h, 26.5 h, 29 h, or 31.5 h p.i. by cervical dislocation. The left lung, the left lateral lobe of the liver, and the entire spleen were harvested and homogenized in 1 mL phosphate-buffered saline (PBS) using a TissueRuptor device (Qiagen). These homogenates were serially diluted and plated on agar plates to determine the number of colonized bacteria in each organ. The bacterial burden was expressed per gram of organ.

### Phylogenetic analysis.

Raw sequencing data for strains Br735, LiA63/2006, LiA161/2005, Jp224, W5 Aug16, and Jp238 are available on the International Pseudomonas Consortium Database (IPCD) and at the National Center of Biotechnology Information (NCBI). The whole-genome sequencing of the other strains was performed on an Illumina MiniSeq (Laboratory of Gene Technology, KU Leuven, Belgium) or NextSeq 500 (Genomics Core, UZ Leuven, Belgium) instrument. Trimmomatic v0.39 was used for read trimming and filtering, including adapter removal, trimming based on quality (LEADING:3; TRAILING:30; SLIDINGWINDOW:4:20), and removal of reads shorter than 36 bases (82). The quality of the reads before and after trimming and filtering was assessed using FastQC v0.11.8. The genomes were assembled *de novo* from the trimmed paired-end reads using SPAdes assembler v3.14.1 (83), and the quality of the draft genome was assessed with QUAST v5.0.2 (84). The genome sequences obtained were annotated with Prokka v1.14.6 using the GenBank compliance option, which removes contigs shorter than 200 bases (85). Core genes, which are present in 99% of the strains, were extracted and analyzed using Roary v3.13.0 with default settings (86). The resulting core gene alignment was used to construct a phylogenetic tree with IQ-TREE v2.1.4 with automatic model selection (87, 88) and ultrafast bootstrapping (UFBoot) (89). Molecular clock rate estimates were generated with TreeTime and used for the construction of an ultrametric tree (90). IToL was used for tree visualization (91).

### Characterization of cytokine responses by quantitative PCR.

After the mice were sacrificed, the inferior lobe of the right lung was collected in RNAlater (Invitrogen). Total RNA was extracted using TRIzol reagent according to the manufacturer’s protocol. In short, samples were thawed and homogenized in TRIzol reagent (Invitrogen) using a Qiagen TissueRuptor device. After addition of chloroform, samples were centrifuged, and the transparent layer was transferred to a new centrifuge tube to which isopropanol was added for RNA precipitation. Samples were then washed with 75% ethanol and air dried for 5 to 10 min. The pellet was resuspended in nuclease-free water, and the concentration was measured using a NanoDrop 2000 spectrophotometer (Thermo Scientific). The extracted RNA was treated with the enzyme ezDNase (Invitrogen) according to the manufacturer’s protocol to remove any remaining genomic DNA. For the cDNA synthesis, a SuperScript IV first-strand synthesis system (Invitrogen) was used following the manufacturer’s protocol. Targets were amplified using oligo(dT) primers to select for mRNA specifically by binding to the poly(A) tail. Both reverse transcriptase (RT) and no-RT control reactions were performed as a control for the presence of remaining genomic DNA during the subsequent PCR. Quantitative PCR (qPCR) was performed using specific primer-probe pairs in three multiplex panels. These panels consisted of Th1-, Th2-, and chemokine-specific pairs. Experiments were performed on a LightCycler 96 (Roche) using the SensiFAST Probe No-ROX kit (Bioline). All data were analyzed with the LightCycler 96-software and normalized by comparing them to the expression of two housekeeping genes (glyceraldehyde-3-phosphate dehydrogenase [GAPDH] and β-actin genes).

### Quantification of tobramycin concentrations in the lungs.

Tobramycin concentrations in the murine lungs were determined via the agar well diffusion method with Bacillus subtilis ATCC 6051 as the indicator strain (92). A standardized inoculum of 10^8^ cells/mL was diluted 1:200 in 1% MHB agar before the agar plates were poured. Holes with a diameter of 8 mm were punched in the solidified agar, and 100 μL of standard tobramycin solution or lung homogenate was introduced into the wells. After overnight incubation at 37°C, the diameters of the inhibition zones were measured with calipers. A standard curve constructed with standard tobramycin solutions, ranging from 0.0625 μg/mL to 32 μg/mL, was used to determine the tobramycin concentration of the lung homogenates. This assay was carried out in triplicate with lungs infected with PA14 from three different mouse experiments, each consisting of two technical repeats.

### Statistical analysis.

All statistical tests were performed on log_10_-transformed data using GraphPad Prism v9.0.0 or R v.4.1.2. When the lowest dilution yielded a CFU count of 0, data points were assigned with a value of half the detection limit. The Shapiro-Wilk test was used to test for normality of the data. Significant changes were assessed through a one-way/two-way analysis of variance (ANOVA) if the variables were normally distributed. In cases of nonnormal distribution, the Kruskal-Wallis test was used. Biphasic nonlinear mixed models were fitted to the time-kill data using the R package nlme based on the following equation: log_10_(survival fraction) = log_10_{[(1 − *P*_0_) × exp(−*k_n_* × *t*)] + [*P*_0_ × exp(−*k_p_* × *t*)]}, where *t* is the treatment time (in hours), *k_n_* and *k_p_* are the killing rates of normal and persister cells (per hour), respectively, and *P*_0_ is the fraction of persister cells (in CFU) at a *t* of 0. The biphasic models were compared with uniphasic nonlinear mixed models (with *P*_0_ = 0) using the Akaike information criterion (AIC). The model with the lowest AIC score indicates a better fit. PGLS analyses using the R packages nlme/ape, caper, phytools, and picante were performed to account for phylogeny when testing the association between survival levels. The constructed ultrametric phylogenetic tree and survival levels were used as input. The determination coefficient (*R*^2^) of the PGLS model was calculated as the *R*^2^_pred_ in the R package rr2 (93).

### Data availability.

Raw sequencing data for strain Br735 are available at NCBI under BioProject no. PRJNA297679 (SRR2939473). Sequencing data for strains LiA63/2006, LiA161/2005, Jp224, W5 Aug16, and Jp238 are available on IPCD (https://ipcd.ibis.ulaval.ca/) and at NCBI under BioProject no. PRJNA325248. Sequencing data of the other strains is available at NCBI under BioProject no. PRJNA940481.
