# Supplementary material for: In Vitro Persistence Level Reflects In Vivo Antibiotic Survival of Natural Pseudomonas aeruginosa Isolates in a Murine Lung Infection Model
Source: Microbiol Spectr. 2023 May 4;11(3):e04970-22. doi: 10.1128/spectrum.04970-22 (PMC10269860; doi:10.1128/spectrum.04970-22)
Supplement: Supplemental file 1 — Supplemental material. Download spectrum.04970-22-s0001.pdf, PDF file, 0.7 MB [file spectrum.04970-22-s0001.pdf]

Supplementary information

***In vitro* persistence level reflects *in vivo* antibiotic survival of natural *Pseudomonas aeruginosa* isolates in a murine lung infection model**

Laure Verstraete[*], Juliana Aizawa[*], Matthias Govaerts, Linda De Vooght, Rob Lavigne, Jan Michiels[†#], Bram Van den Bergh[†], Paul Cos[†]

[*] These authors contributed equally to the work as first authors

[†] These authors contributed equally to the work as senior authors

[#] Corresponding author: jan.michiels@kuleuven.be

This PDF file includes:

Supplementary Tables S1 to S3

Supplementary Figures S1 to S8

Supplementary tables

Table S1: Dose-response experiments to determine the inoculum dose. Two mice per inoculum were infected with *P. aeruginosa* strain PA14 embedded in seaweed alginate beads at different inoculum doses. The mortality rate was recorded over 48 h.

CFU per mouse	Mortality at 48 h p.i.
1×10^8	100%
1×10^7	100%
5×10^6	100%
1×10^6	50%
5×10^5	0%
1×10^5	0% (clearance)

Table S2: Estimates of *in vitro* survival fraction based on biphasic kill fits

Strain	Estimate of log_{10}(survival fraction)	95% confidence intervals
Br257	-4.51	[-4.01,5.02]
Br735	-5.68	[-5.18,-6.18]
Br817	-5.29	[-4.60,-5.97]
BSM70-467	-4.38	[-3.66,-5.11]
C13	-5.91	[-5.34,-6.47]
GCF85-227	-1.05	[-0.07,-2.03]
IC12	-4.62	[-4.07,-5.16]
Jp1504	-4.53	[-3.95,-5.10]
Jp224	-6.57	[-6.05,-7.10]
Jp238	-3.45	[-2.86,-4.03]
LiA11/2004	-4.30	[-3.75,-4.85]
LiA141/2007	-3.64	[-3.01,-4.26]
LiA161/2005	-5.37	[-4.87,-5.87]
LiA63/2006	-5.02	[-4.38,-5.67]
LiA86/2007	-6.14	[-5.56,-6.71]
LMG 1272	-6.16	[-5.59,-6.74]
Lo050	-1.60	[-1.04,-1.60]
W5 Aug16	-3.79	[-3.21,-4.37]
PA14	-5.35	[-4.77,-5.93]

Table S3: Comparison of the OLS model and PGLS model. SE = standard error, CI = confidence interval, AIC = Akaike Information Criterion

regression model	λ/κ	intercept	SE	95% CI	p-value	slope	SE	95% CI	p-value	R^2	AIC
OLS	0	0.1168	0.4988	[-0.9355,1.1691]	0.8177	0.2691	0.1037	[0.0504,0.4879]	0.01880	0.2417	40.813
PGLS	1	-0.4302	1.9667	[-4.5673,3.7068]	0.8294	0.1288	0.0465	[0.0307,0.2269]	0.01309	0.2491	86.118

Supplementary figures

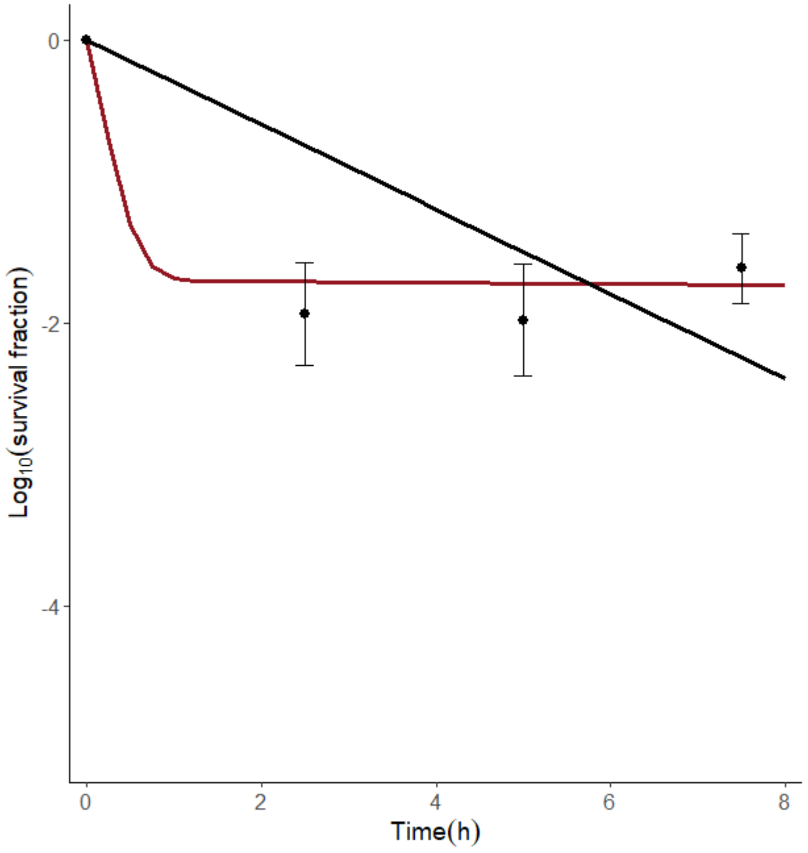

Figure S1: Time-kill curve of the PA14 murine lung population after tobramycin treatment*.* *Both a biphasic exponential curve (in red) and an uniphasic exponential curve (in black) were fit to the time-kill data of Figure 1. Based on the Akaike Information Criterion (AIC) values, the biphasic model is superior over the uniphasic model to describe the observed killing dynamics.*

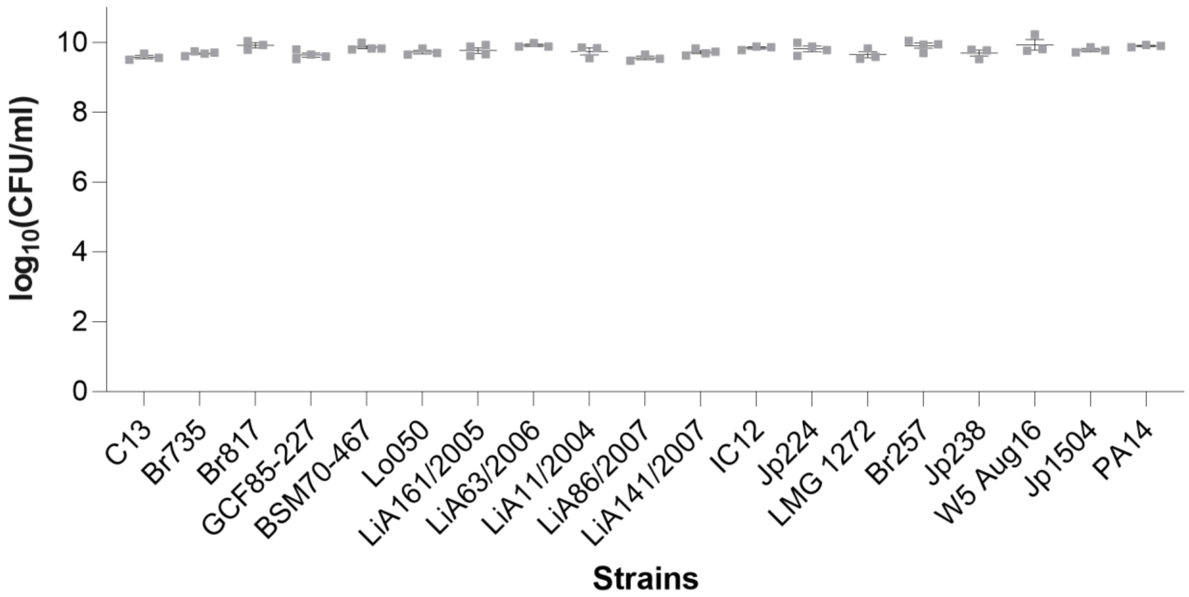

Figure S2: Total number of cells at the start of* in vitro *time kill assays. Overnight cultures were 1:100 diluted in fresh medium and incubated for 16h. Before the start of the antibiotic treatment, CFUs were determined by plate counting. The number of cells were similar between the different strains and range from 3.2 x 10^9 to 1.68 x 10^{10} CFU/ml, demonstrating no clear differences in *in vitro* fitness.

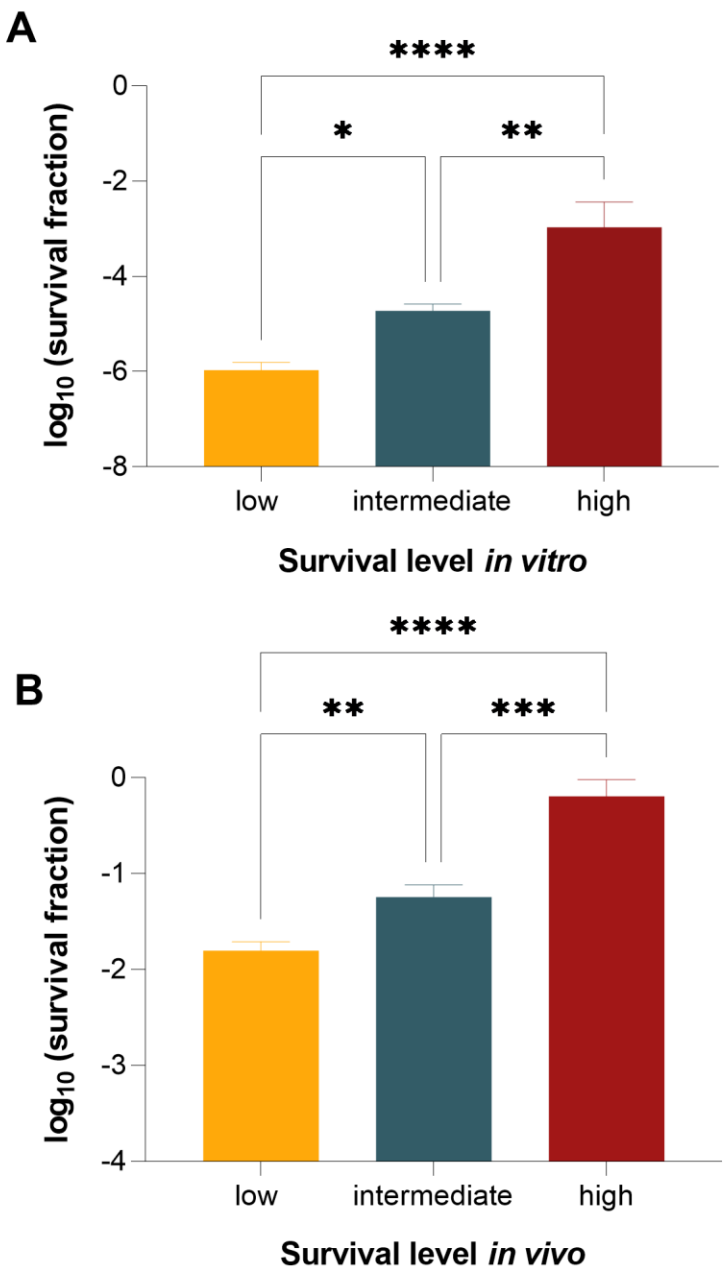

Figure S3: The survival fraction between the three survival groups is significantly different. *Bars are colored according to their survival level* in vitro *(A) and* in vivo *(B). Error bars show the standard error of the mean. Statistical differences between the* in vitro *groups are determined with one-way ANOVA followed by Tukey's* post hoc *test for multiple comparisons. Statistical differences between the* in vivo *groups are determined via Kruskal-Wallis test followed by Dunn's* post hoc *test for multiple comparisons. *, P < 0.05; **, P < 0.01; ****, P < 0.0001.*

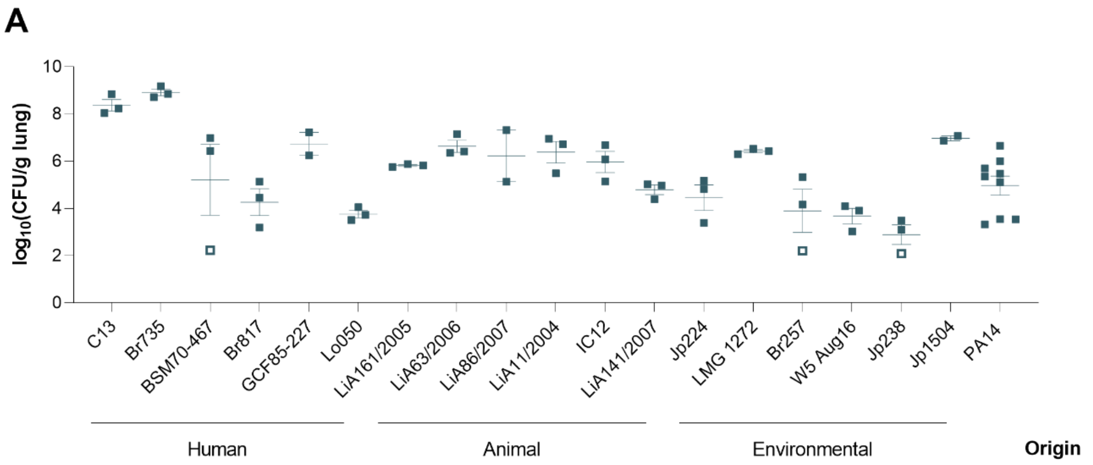

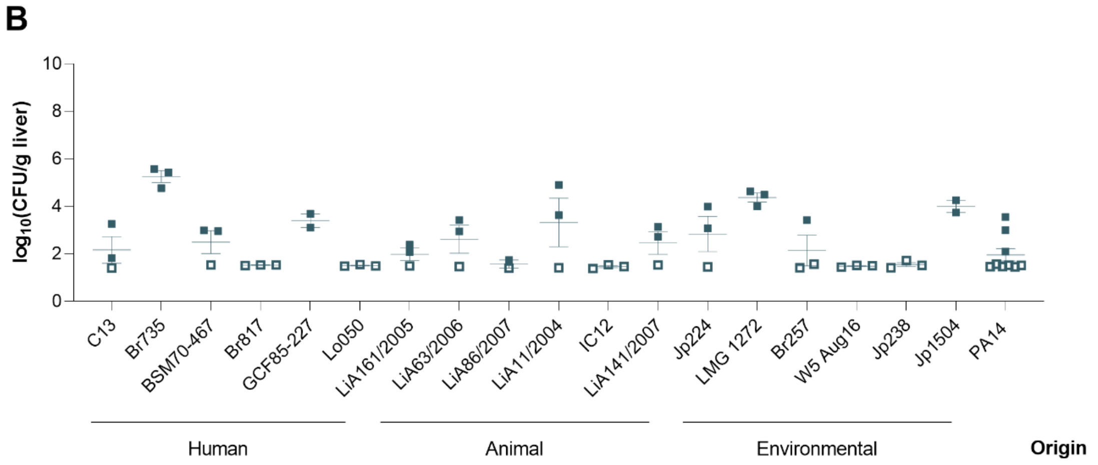

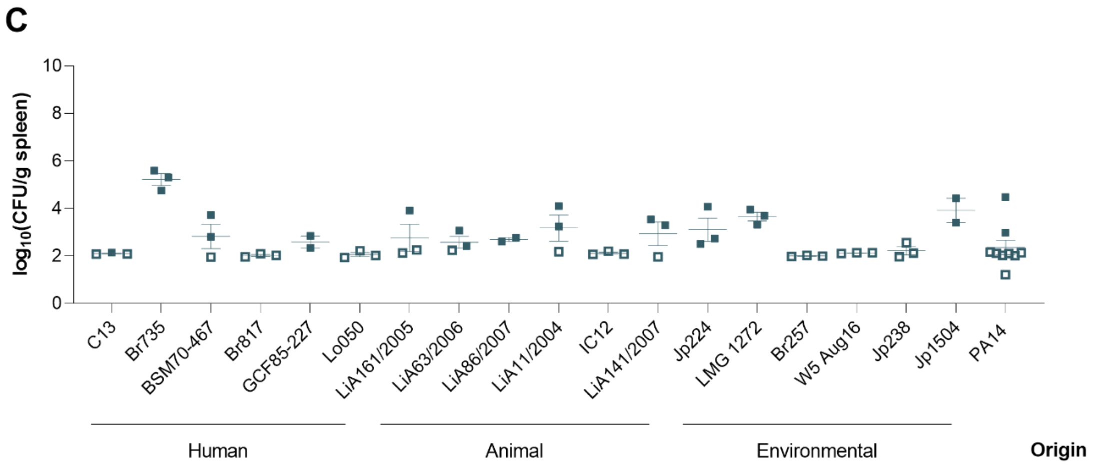

Figure S4: Bacterial load in the left lung (A), liver (B) and spleen (C) at 26.5 h p.i. of untreated mice. *Open squares indicate repeats below the detection limit of which half of the detection limit divided by the organ weight is shown. Error bars represent the standard error of the mean.*

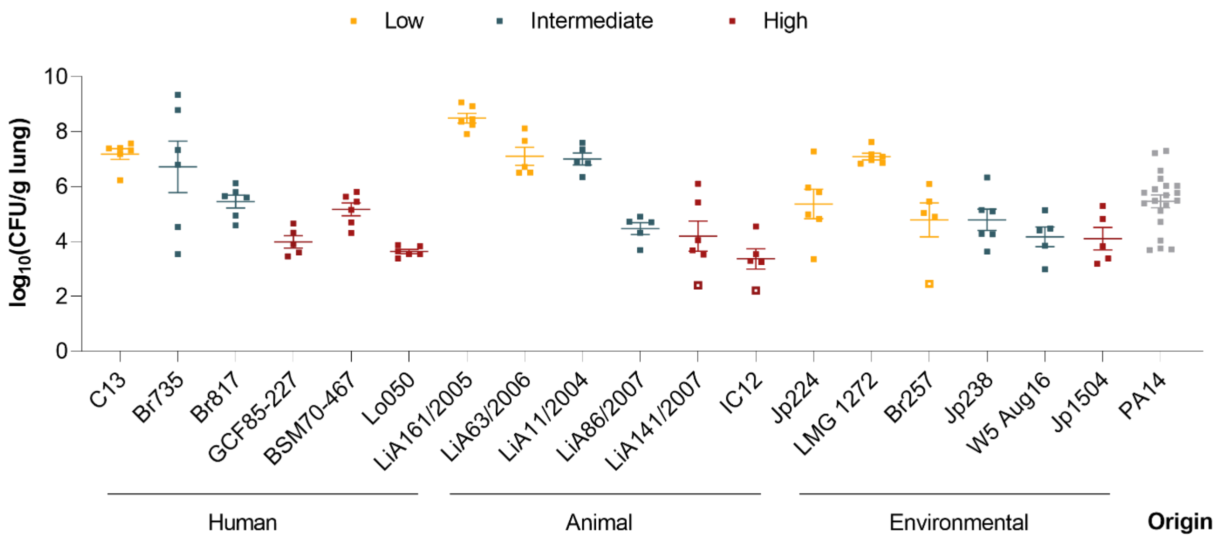

Figure S5: Bacterial counts of untreated mice at 26.5 h p.i. *Strains are sorted according to their origin and colored according to their* in vivo *survival phenotype with yellow, blue, and red representing, respectively, low, intermediate and high survival levels after antibiotic treatment. Each square represents the CFU count of untreated mice at 26.5 h p.i. Open squares indicate untreated samples below the detection limit. Error bars represent the standard error of the mean. CFU counts were compared between strains using one-way ANOVA showing a significant difference in number of bacterial cells ($p < 0.0001$).*

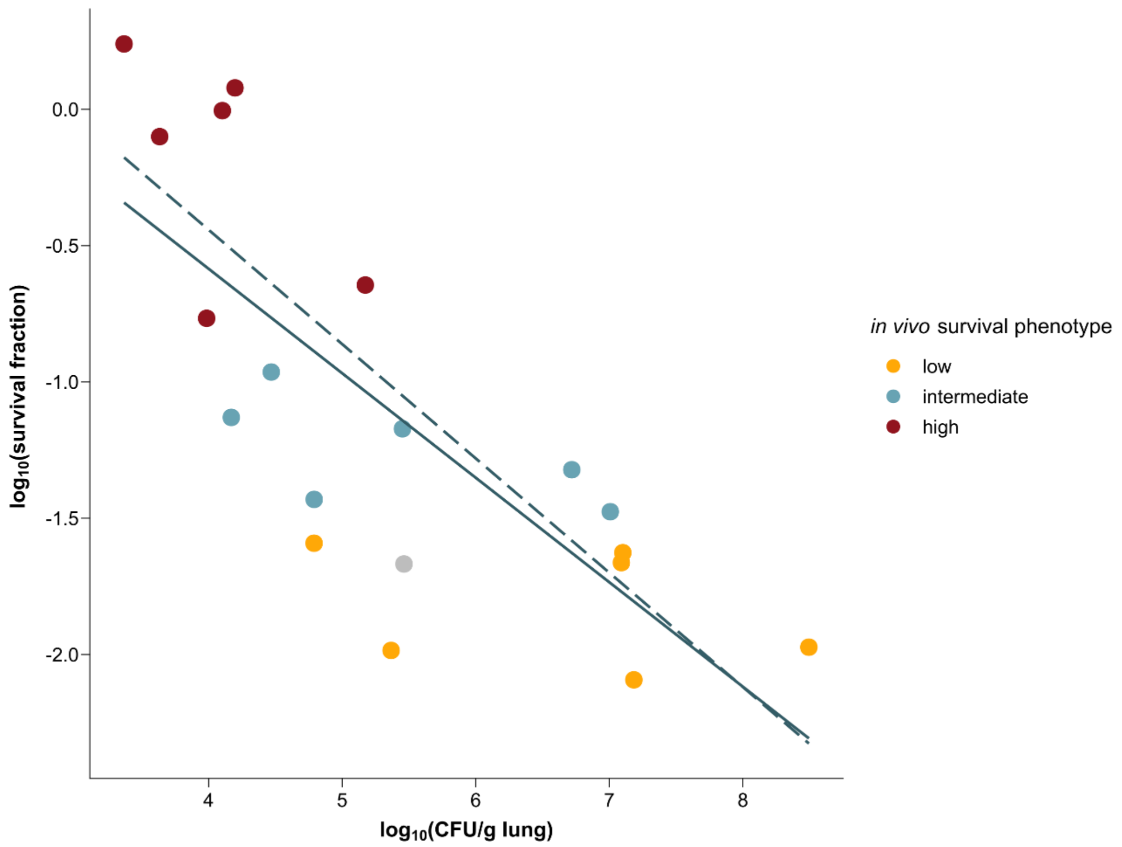

Figure S6: **In vivo *survival is negatively correlated with* in vivo *fitness.*** *The correlation between the* in vivo *survival fraction (shown in Figure 4) and the CFU counts of untreated mice at 26.5 h p.i. (shown in Figure S5) is shown for all tested* P. aeruginosa *isolates. The 18 natural isolates are colored according to their phenotype* in vivo. *The lab strain PA14 is represented by the grey dot. The solid line shows the OLS regression line and the dashed line the PGLS regression line. The* R^2 *of the OLS and PGLS model is 0.56 and 0.57, respectively (*$p < 0.0001$*).*

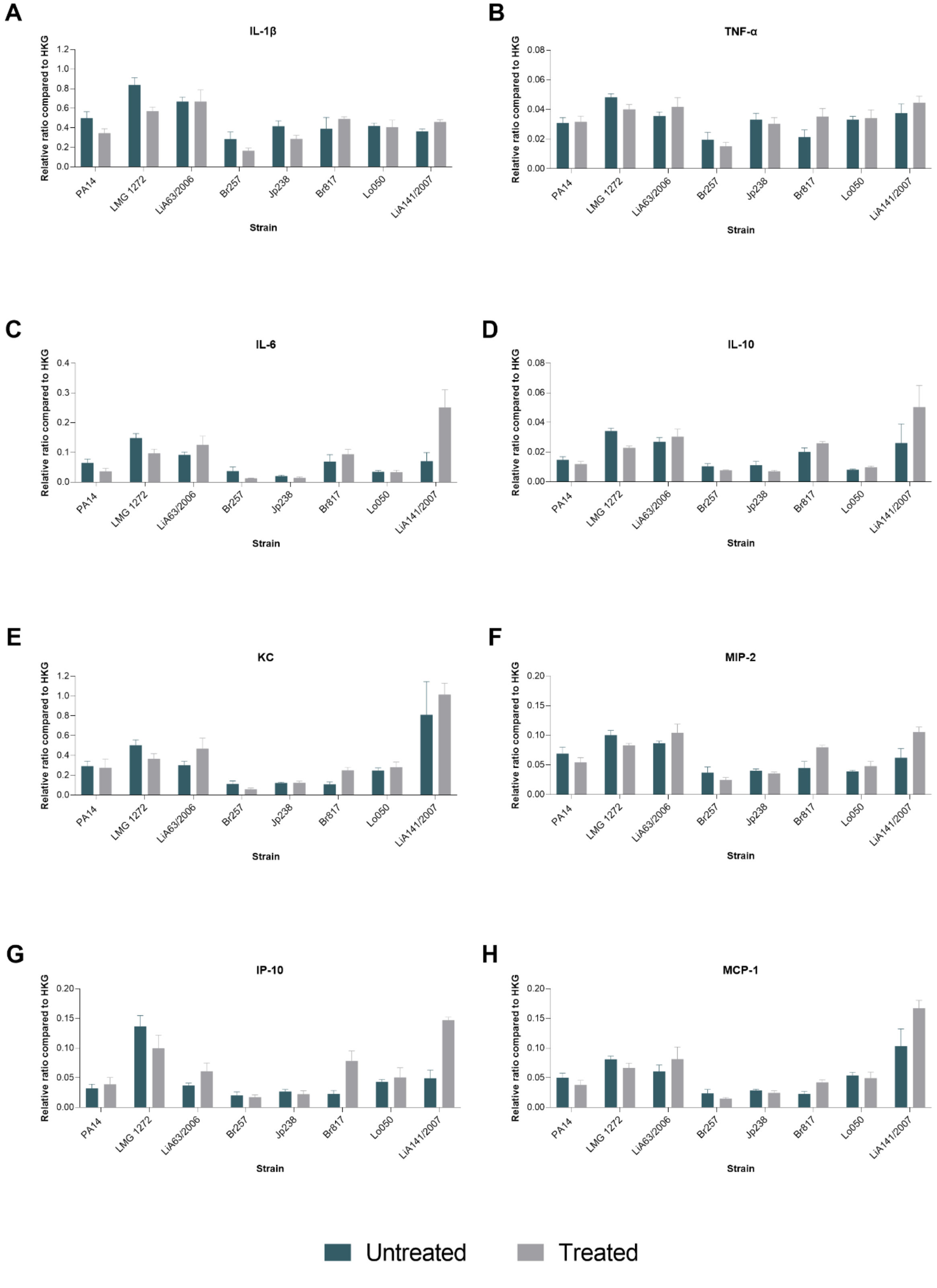
A
IL-1β
B
TNF-α
C
IL-6
D
IL-10
E
KC
F
MIP-2
G
IP-10
H
MCP-1
Relative ratio compared to HKG
Strain
PA14
LMG 1272
LiA63/2006
Br257
Jp238
Br817
Lo050
LiA141/2007
Untreated
Treated

Figure S7: RT-qPCR analyses on a selection of isolates to determine the cytokine and chemokine expression levels. *Quantitative PCRs were performed on the right lung of mice (5-6 mice per group) infected with different* P. aeruginosa *isolates. The mice were sacrificed 26.5 h p.i. with or without tobramycin treatment as indicated by the colours. Strains are sorted according to their* in vivo *survival, PA14 has the lowest survival and LiA141/2007 the highest survival. Expression levels are expressed relative to the expression of two housekeeping genes (GAPDH and β-actin). Except for IP-10, there is no significant difference in inflammatory response upon antibiotic treatment. Statistical analyses were performed per cytokine/chemokine with two-way ANOVA. Error bars represent the standard error of the mean.*

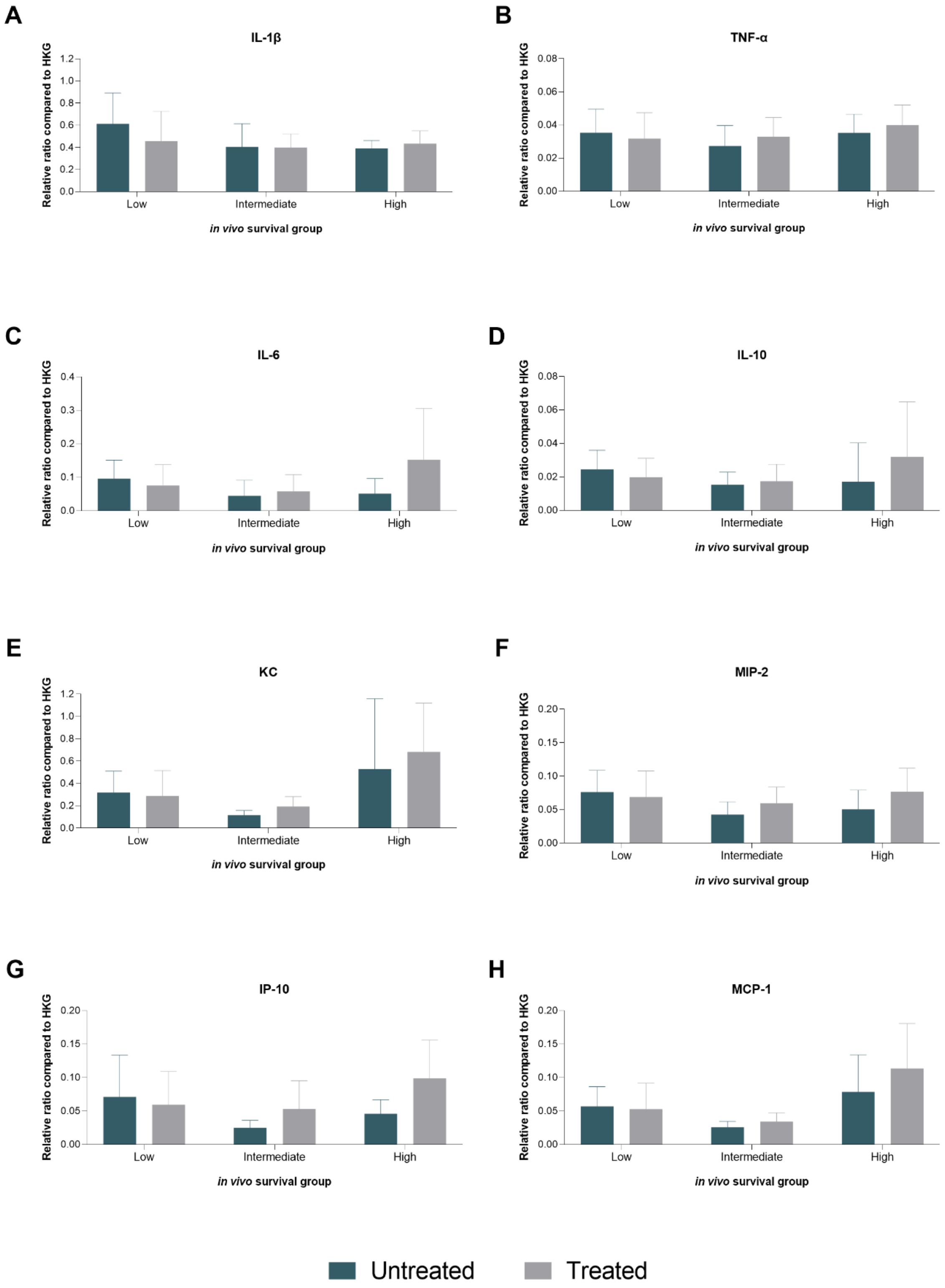

A
IL-1β
Relative ratio compared to HKG
1.2
1.0
0.8
0.6
0.4
0.2
0.0
Low
Intermediate
High
in vivo survival group
B
TNF-α
Relative ratio compared to HKG
0.08
0.06
0.04
0.02
0.00
Low
Intermediate
High
in vivo survival group
C
IL-6
Relative ratio compared to HKG
0.4
0.3
0.2
0.1
0.0
Low
Intermediate
High
in vivo survival group
D
IL-10
Relative ratio compared to HKG
0.08
0.06
0.04
0.02
0.00
Low
Intermediate
High
in vivo survival group
E
KC
Relative ratio compared to HKG
1.2
1.0
0.8
0.6
0.4
0.2
0.0
Low
Intermediate
High
in vivo survival group
F
MIP-2
Relative ratio compared to HKG
0.20
0.15
0.10
0.05
0.00
Low
Intermediate
High
in vivo survival group
G
IP-10
Relative ratio compared to HKG
0.20
0.15
0.10
0.05
0.00
Low
Intermediate
High
in vivo survival group
H
MCP-1
Relative ratio compared to HKG
0.20
0.15
0.10
0.05
0.00
Low
Intermediate
High
in vivo survival group
Untreated
Treated

Figure S8: Cytokine and chemokine expression levels following* P. aeruginosa *infection classified per survival group. *The same expression levels as Figure S4 are presented here, but are now shown per* in vivo *survival group instead of per strain. Strains are sorted according to their* in vivo *survival group, with the low survival group representing strains with the lowest survival fraction* in vivo *(LMG 1272, LiA63/2006 and Br257) and the high survival group representing strains with high survival fractions* in vivo *(Lo050 and LiA141/2007). The strains Jp238 and Br817 belong to the intermediate survival group. The lab strain PA14 was not included here for data analysis. The expression levels of KC and MCP-1 are significantly higher in the high survival group compared to the two other survival groups. The expression level of MIP-2 was highest in the low survival group. No significant differences between survival groups are observed for the other cytokines/chemokines. Statistical analyses were performed per cytokine/chemokine with two-way ANOVA and Šídák's* post hoc *test for multiple comparisons. Error bars show the standard deviation.*
